# Journal of Clinical Monitoring and Computing 2017/2018 end of year summary: monitoring—and provocation—of the microcirculation and tissue oxygenation

**DOI:** 10.1007/s10877-019-00270-7

**Published:** 2019-02-11

**Authors:** J. J. Vos, S. F. Ellermann, T. W. L. Scheeren

**Affiliations:** Department of Anesthesiology, University of Groningen, University Medical Center Groningen, Hanzeplein 1, PO Box 30.001, 9700RB Groningen, The Netherlands

**Keywords:** Monitoring, Tissue oxygenation, Microcirculation, NIRS, Vascular occlusion test, Visualization

## Abstract

The microcirculation is the ultimate goal of hemodynamic optimization in the perioperative and critical care setting. In this fourth end-of-year summary of the Journal of Clinical Monitoring and Computing on this topic, we take a closer look at papers published in the last 2 years that focus on this important aspect. The majority of these papers investigated the use of either cerebral or peripheral tissue oxygen saturation, derived non-invasively using near infrared spectroscopy (NIRS). In some of these studies, the microcirculation was “provocated” by inducing short-term tissue hypoxia, allowing the assessment of functional microvascular reserve. Additionally, studies on technical differences between NIRS monitors are summarized, as well as studies investigating the feasibility of NIRS monitoring, mainly in the pediatric patient population. Last but not least, novel monitoring tools allow assessing oxygenation at a (sub)cellular level, and those papers incorporating these techniques are also reviewed here.

## Introduction

This is already the fourth Journal of Clinical Monitoring and Computing end-of-year review that is contributed to monitoring the microcirculation and tissue oxygenation in the perioperative setting within 5 years. While there is still a reasonable number of papers dedicated to these research areas, a gap between routine clinical perioperative care and implementation of microcirculatory monitoring still exists. This review aims to provide a comprehensive summary of relevant recently published papers in this journal in the past two years.

Hemodynamic monitoring and management are mainly focused on optimizing macro-hemodynamic variables (e.g. blood pressure, heart rate, stroke volume, cardiac output), of which the ultimate goal is to ensure oxygen delivery and optimal microvascular conditions in important organs, such as the heart, brain and kidneys. The microcirculation is a key component of the cardiovascular system as it facilitates nutrient and gas exchange at the tissue level. It consists of blood vessels with diameters below 150 µm, making up a dense network of arterioles, capillaries and post-capillary venules. Local and systemic factors regulate blood flow by modulation of precapillary sphincters, with perfusion taking place depending on the tissue’s metabolic need. With the exception of near-infrared spectroscopy (NIRS), which allows monitoring of tissue oxygen saturation, assessing the condition of “individual” tissues or organs in the perioperative phase in a continuous and least-invasively manner is more cumbersome, time-consuming and hence is generally used for investigational and experimental purposes. It may therefore not be surprising that most papers summarized here investigate the use of some form of NIRS for microcirculatory monitoring. Hence, the first part of this summary will deal with these NIRS-related papers. The second part of this summary will deal with other, novel and promising monitoring techniques that allow assessing oxygen delivery at a (sub)cellular level.

## Near infrared spectroscopy

NIRS provides a functional imaging modality, in which near-infrared light is used to noninvasively assess microvascular changes in local oxy- and deoxyhemoglobin saturation. The ratio between these saturations reflects local oxygen saturation of the tissue of interest (StO_2_). Most commonly, NIRS is used for cerebral oxygenation monitoring, and its use is widespread during adult or pediatric cardiothoracic surgery, and in neonates undergoing surgery or during their stay at the neonatal intensive care unit.

There are different NIRS monitors commercially available [1] that allow monitoring tissue oxygenation either cerebrally or peripherally. The general principle, i.e. assessing oxygenation using the Lambert–Beer law, is an equally shared principle for all NIRS monitors [2]. However, as we will see later on in this review, there are important technical differences between these monitors, mainly based on the handling of light scatter by the underlying algorithms and the fixed ratio of weighting the contribution of arterial and venous blood to the signal.

An important consideration in the interpretation of NIRS-related variables, both for cerebral oxygen saturation (ScO_2_) and (peripheral, or “somatic”) StO_2_, is the accordance of the obtained readings with reference values. In the April 2018 issue of this journal, Benni et al. [3] give us insight into the validation method for the FDA of a NIRS-based oximeter for both cerebral and somatic tissue oxygenation measurements in adult subjects. For cerebral validation, forehead ScO_2_ measurements were compared to a weighted reference of internal jugular bulb and arterial blood oxygen saturations of 25 healthy adult volunteers of different skin tone during a controlled hypoxia sequence. For somatic validation, three sensors were placed around muscle tissue on the flank, thigh and calf, and compared to similarly weighted oxygen saturations derived from arterial and central venous blood. Hypoxia was induced by stepwise reduction of inspiratory oxygen concentration until pulse oximetry reached an oxygen saturation (SpO_2_) of 70%, while maintaining end-tidal carbon dioxide (CO_2_) levels at 40 ± 2 mmHg to control cerebral vasoreactivity. The accuracy of ScO_2_ measurements of both hemispheres was similar, although jugular bulb measurements were always performed on the right side. With a mean difference of 30 ± 6% between arterial and jugular bulb oxygen saturation, changing the weighting ratio of the contribution of venous and arterial blood to brain tissue oxygenation from 60:40 to 80:20% resulted in a bias change of ± 3% compared to a fixed ratio of 70:30%, while skin tone did not affect the results. It should be mentioned that these results were obtained at a fixed blood CO_2_ level, limiting cerebral vasoreactivity, and that results might be less accurate under normal clinical conditions. For the somatic measurements, accuracy of tissue oxygenation measurements was lower than for the cerebral values, and it further decreased as the somatic measurement location was farther away from the central venous blood draw location. A reason for this might be the varying ratios between venous and arterial blood contribution to the signal, e.g. with body position (blood pooling) or muscle activity (perfusion and metabolism). The use of a standardized validation protocol as suggested in this study might contribute to increase the reliability and consistency of NIRS measurements obtained with tissue oximeters from different manufacturers, as has been achieved with pulse oximeters years ago.

Another method comparison study on NIRS devices was published by Ferraris et al. [4] in the same issue. Although such devices are primarily developed for cerebral oximetry, the authors compared peripheral (somatic) StO_2_ values obtained on the forearm with two different last generation four-wavelength NIRS devices in 20 adult patients undergoing elective cardiac surgery at several predefined time points. No significant differences and a good correlation were found between the StO_2_ values of both devices with a bias of 4%, however with limits of agreement of ± 26%. The authors conclude that regarding absolute StO_2_ values both devices were equivalent, but that the clinical agreement was not acceptable, so that both devices are not interchangeable in daily practice.

### “Provocation” of the microcirculation and NIRS

Bedside monitoring of tissue oxygenation provides a unique opportunity to dynamically assess functional microcirculatory reserve and to allow specification of local oxygen consumption and vascular reactivity. Most commonly, this is achieved by performing a vascular occlusion test (VOT), but as we will see, other more experimental techniques (arterial clamping, induced hypoxemia) are also available.

#### Provocation by vascular occlusion test

**Fig. 1:**
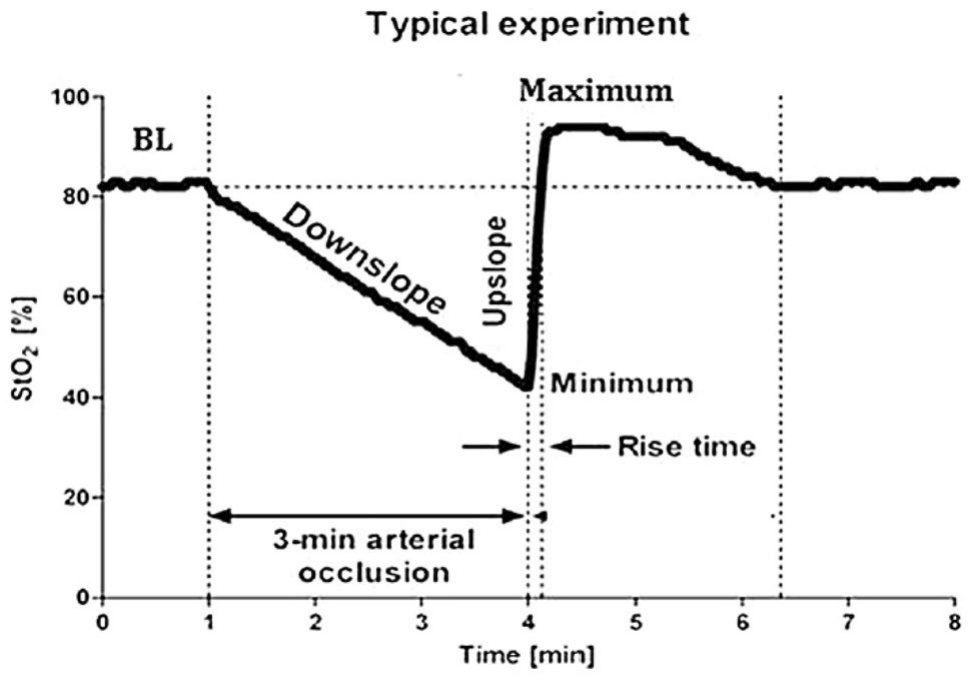
Representative example of changes in tissue oxygen saturation (StO_2_) induced by performing a vascular occlusion test (Reprinted with permission from Steenhaut et al. [6])

The VOT remains a popular test to assess—by provocation—the microcirculatory response to sudden ischemia while measuring StO_2_. For performing a VOT, a tourniquet is placed proximal to the measurement site and inflated above systolic blood pressure for 3–5 min, blocking both arterial inflow and venous outflow of the site of interest. The resulting slopes following initiation and release of VOT—of which Fig. 1 serves as an example—can be divided in a down- and upslope part. The initial downslope of the VOT curve gives an impression of local muscular oxygen consumption, i.e. (local) VO_2_. The second upslope curve *after* deflation of the cuff gives an impression of the adequacy of reperfusion. Details on the VOT curve have extensively been discussed previously [5]. Performing a VOT is not a very well standardized technique, with important variation in for example the actual duration of tourniquet inflation, as this can be done either in a fixed time window (e.g. 3 or 4 min) or in a variable time-window while using a lower StO_2_ cut-off value (usually 40%) for releasing cuff pressure.

In the December 2017 issue of this journal, it was hypothesized by Steenhaut et al. [6] that despite differences in measurement techniques between different NIRS monitors, StO_2_ trends induced by a VOT should be similar among these devices. The authors performed VOT’s in patients scheduled for coronary artery bypass surgery (n = 40) and measured StO_2_ using three devices (INVOS, Foresight, NIRO-200NX) from three manufacturers (Covidien, Casmed and Hamamatsu, respectively). Unfortunately, their hypothesis was rejected, and except for most *static* VOT characteristics (e.g. baseline StO_2_, before initiation a VOT), all observed *dynamic* characteristics (both the down- and upslope part of the VOT) turned out to be different. Similarly, the study of Ferraris et al. [4] discussed above found significant differences from individual baseline StO_2_ values during a VOT between the two devices studied (Masimo O3 and Nonin EQUANOX, respectively). These clear observations indicate that differences in NIRS techniques itself (e.g. the number of wavelengths used, tissue penetration depth) and possibly differences in the computation algorithms, yield important monitor-specific differences in VOT characteristics, and these differences should be acknowledged in future studies.

Apart from the technical differences between NIRS devices that likely influence characteristics of the VOT-derived variables, multiple patient-related factors have been identified that further influence the characteristics of the VOT curve, e.g. sepsis [7]. Importantly, induction of general anesthesia itself is known to influence the microcirculation and possibly, anesthesia-related changes to VOT variables should also be considered. In a randomized study by Cho and co-workers published in the October 2017 issue of this journal [8], the differences in VOT characteristics between propofol-remifentanil and desflurane-remifentanil anesthesia were investigated in patients undergoing thoracoscopic surgery. The authors reported a consistently higher StO_2_ (measured at the thenar eminence by the Inspectra™ 650 device, Hutchinson, USA) in the desflurane group at multiple time periods during surgery, together with a subtly increased recovery slope in this group. The authors state desflurane-remifentanil anesthesia to be associated with “better” preserved microcirculation compared to propofol-remifentanil anesthesia. These results should be interpreted with some caution, as the authors only studied one surgical procedure (thoracoscopic surgery) and it is unclear whether the propofol-remifentanil and desflurane-remifentanil group received an equipotent depth of anesthesia. Moreover, the meaning of the subtle difference in recovery slope (0.5%) for clinical outcome is unknown. Nevertheless, the paper touches an important aspect frequently neglected in clinical papers: the differences in hemodynamics and microcirculation induced by the use of multiple, different anesthetics and their hemodynamic influences. Obviously, future studies should provide further elucidation of these important characteristics of our frequently used drugs in perioperative and intensive care medicine.

The association of VOT-derived variables and “outcome” was the focus of a recent paper by Nam et al. published in April 2018 in this journal [9]. The authors associated the recovery slope of the VOT—performed at skin closure following cardiac surgery—with the amount of postoperative chest tube output and need for RBCs. For both variables, an inverse and independent relation was found. The authors postulate that this finding elucidates the role of the endothelial cells—being an important part of the microvasculature—in regulation of endothelial coagulation. Indeed, as these data show, the provocation of the microvasculature by applying a VOT provides an “impression” of its functional state.

In another study by Tujjar in the June 2017 issue of this journal [10], the evolution of StO_2_ following a rather extensive “VOT” was analyzed. First, for patients who underwent extremity surgery, regional anesthesia was provided, which was associated with a rise in StO_2_. Subsequently, a tourniquet was applied for creating bloodless conditions up to durations of 30, 60 and 90 min. Interestingly, the authors observed that recovery characteristics following desufflation of the tourniquet were correlated with the length of ischemia—not with patient demographics—and were more pronounced for the longer procedures (i.e. 90 min), with a decrease in the upslope, hyperemic peak and longer recovery time. The authors postulate that these changes merely reflect reductions in microvascular blood flow following prolonged ischemia, rather than changes in metabolic rate, given that regional anesthesia was applied.

#### Experimental provocation by aortic clamping

In a substantially different setting, spinal cord ischemia was induced experimentally in swine, in order to assess monitoring of spinal cord perfusion using NIRS. In this study, published in the October 2017 issue by Suehiro et al. [11], aortic cross-clamping was performed distally to the subclavian artery, and NIRS electrodes were placed along the midline of the middle and lower thoracic vertebrae. Expectedly, NIRS values dropped after clamping, and were restored to normal values after declamping, 30 min later. The interesting point in this paper is that the authors additionally drained liquor, which is frequently performed in clinical practice for optimizing spinal perfusion pressure when perfusion is presumed at risk, such as during surgery for thoracic aneurysm repair. The authors were unable to identify any difference in NIRS-values after initiation of liquor drainage. This might suggest that either perfusion was not optimized per se, by lowering spinal pressure, or otherwise that NIRS photons do not penetrate that far that such (presumed) subtle difference can be noticed. Ultimately, this finding might suggest that rather than the spinal cord itself, oxygenation of the overlying tissues was monitored.

#### Provocation by experimentally controlled hypoxia

This latter issue touches a highly relevant issue: the level of superficial “admixture” from superficial tissue for monitoring of the deeper lying “target” organ. Importantly, most NIRS devices that are used clinically resolve their data using continuous-wave (CW) techniques, primary based on the Lambert–Beer principle [2]. The key limitation of this technique is the uncertainty of resolving light from specific depths, e.g. superficial tissue “admixture” might occur. Spatially resolved spectroscopy (SRS) further augments CW-NIRS and provides some means to improve depth resolution, whereas frequency domain (FD) NIRS—in which light is modulated at particular frequencies to allow derivation of tissue-specific values—proposes another NIRS modality. The technical differences between CW- and FD-NIRS are beyond the scope of this review, but are excellently described in this journal in October 2017 by Davies et al. [12]. In this paper, controlled isocapnic hypoxia was induced in nine healthy volunteers to elucidate differences between SRS-CW- and FD-NIRS monitoring devices. The authors determined both cerebral (intracranial) and zygomal (extracranial) oxygen saturation using two NIRS monitoring devices that are commercially available for bedside monitoring. Interestingly, the authors documented indistinguishable recording of both cerebral and extracranial tissue oxygen saturation for SRS-CW- and FD-NIRS, albeit the latter had more “noise”. Although these data are derived in a small study, it suggests no clear benefit from using more complex NIRS monitors incorporating FD-techniques for clinical purposes.

A recent case-report by Lanks et al. in the 2018 August issue of this journal [13], describes the evolution of SRS-CW obtained cerebral oxygenation saturation before occurrence of an in-hospital cardiac arrest in an adult critically ill patient treated with four vasopressors for severe shock. This patient had a cerebral tissue oxygen saturation of only 40% in the time period preceding circulatory arrest, which dropped precipitously in the 15 min preceding cardiac arrest, whilst macro-hemodynamic variables—i.e. mean arterial blood pressure (MAP)—remained ‘stable’ in that period and showed no sign of deterioration. Hence, NIRS-derived variables may have functioned as an early warning sign here, and maybe it might be used as such.

## NIRS-derived variables and outcome

NIRS-derived regional or cerebral oxygen saturation potentially allows assessment of outcome and/or guiding care and the use of hospital resources. This issue was assessed in this journal in February 2018 by Balakrishnan et al. [14] in pediatric ICU patients with respect to the requirement for lifesaving interventions in the first 24 h following ICU admission. The use of such interventions, e.g. fluid resuscitation, intubation, and emergency surgery, was more prevalent in pediatric patients with NIRS-derived somatic oxygen saturation < 70%, as shown in this retrospective chart study of 411 patients. Importantly, the 70% cut-off was predefined, and was together with an age-corrected increase in heart rate, the only variable that was significantly associated with the use of lifesaving interventions. Additionally interesting was that the association between the use of life saving interventions and cerebral oxygen saturation was less prominent, even though cerebral oxygen saturation tended to be lower in patients with somatic oxygen saturation < 70%. Importantly, cerebral oxygen saturation was monitored in fewer patients—and thus a smaller applicable sample size was obtained allowing less definitive conclusions, this observation still suggests that cerebral autoregulation maintained cerebral oxygenation in these patients, in spite of regional oxygen saturation at the somatic site of measurement, the posterior flank.

Cerebral oximetry is and remains the major application of the NIRS technology, reflecting the balance between oxygen delivery and consumption in the brain. Although the main application remains the cardiosurgical setting, other fields of surgery are gaining interest in this technology as well. In the December 2018 issue of the journal, Clemmesen and coworkers [15] published the results of an observational pilot study in which they prospectively explored the association between cerebral oxygenation (ScO_2_) and postoperative mortality and delirium in 40 elderly (age > 65 years) patients with hip fracture. These outcomes occurred in 10% and 25% of patients, respectively, which are rather small cohorts difficult to draw firm conclusions from. Nevertheless, there were significantly lower and a non-significant trend for lower admission ScO_2_ values in non-survivors and in patients who developed postoperative delirium, respectively. 30-day-mortality was significantly associated with low admission ScO_2_ values (< 55% for at least 2 min), but not with low blood pressure (defined as MAP < 60 mmHg). In addition to the initial resuscitation period after admission, ScO_2_ was also monitored intraoperatively during hip surgery in 26 of the 40 patients (65%). These intraoperative ScO_2_ values were significantly lower than those at the preoperative resuscitation period (60.5 vs. 65.5%, p < 0.001), and tended to be lower in non-survivors and patients developing postoperative delirium, although due to the small numbers none of these subgroup differences were statistically significant. Taken together, these results underline the importance of regional perfusion measures such as ScO_2_ as opposed to systemic hemodynamic values, and that these measurements should be extended over the whole perioperative period in this population of frail patients with multiple comorbidities.

Another study by Li and co-workers [16]—published in April 2018 in this journal—performed in hypertensive patients undergoing major abdominal surgery, investigated the incidence of intraoperative cerebral oxygen desaturation, and determined its association with changes in cognitive function tests. The attending anesthesiologists were blinded for cerebral oxygenation readings during anesthesia. The results were remarkable: of the 41 patients studied, 20 patients had an intraoperative desaturation of cerebral oxygenation, defined as a reduction > 20% from their awake baseline values. In addition, the proportion of patients that showed a reduction in cognitive function tests 4 days postoperatively was increased in those that had an intraoperative cerebral desaturation. Importantly, there were no differences in either baseline or intraoperative values of blood pressure between both groups, whilst in the group of patients that showed cerebral oxygenation desaturations, more patients had an uncontrolled hypertension (according to whether blood pressure targets were achieved pre-operatively) and had a higher hypertension risk-stratification, as based on factors such as end-organ damage. Though this was only a small sized pilot study, it highlights two important issues: at first, it shows that in the investigated population, a substantial number of patients exhibit intraoperative cerebral oxygen desaturation, that would have gone unnoticed if it weren’t monitored. It remains to be proven whether therapeutic decisions based on preserving cerebral oxygen saturation, such as titrating fluids, vasopressors and inotropes, would have been helpful. Secondly, the study demonstrates the association between intraoperative cerebral tissue desaturation and a postoperative reduction in cognitive function. This observed difference in outcome underlines the importance of monitoring end-organ dynamics during general anesthesia, and urges the need for high quality trials aimed at assessing whether optimization of, for instance cerebral oxygenation, improves (organ-related) outcome.

In another study published in October 2018 in this journal by Feng Jin et al. [17], unilateral ganglion stellate blockade (GSB) was performed, aimed to investigate its effect on sleep quality in postoperative patients following breast cancer surgery. While this study raises the suggestion that performing GSB might have contributed to a slightly improved quality of sleep, there were no differences in cerebral oxygen saturation observed. This observation contrasts a previous report [18] that showed an ipsilateral increase, and contralateral decrease in cerebral oxygen saturation following GSB, so further investigations are warranted to investigate whether the postulated effects of GSB on cerebral blood flow (i.e., an increase in the blocked side) are actually present or not. In addition, the interplay between cerebral metabolism and perfusion under awake circumstances in a rather broad patient population is complex and likely based on multiple factors, for which it might be hard to easily discriminate direct regional changes in perfusion secondary to GSB.

Manipulation of abdominal organs during surgery can cause a mesenteric traction syndrome (MST), which presents with hypotension, tachycardia and flushing, probably mediated by release of prostaglandins. In the April 2018 issue, Olesen and co-workers [19] investigated whether MST also affects ScO_2_ measurements, and if this was caused by an increase in forehead skin blood flow. They included 22 patients undergoing major upper abdominal surgery, 10 of which presented with MST intraoperatively. Patients with MST had an increased heart rate, cardiac output, ScO_2_ and skin blood flow, but similar blood pressures and middle cerebral artery flow velocity obtained by transcranial Doppler than those without MST. These results show that increases in ScO_2_ were exclusively caused by increases in extracranial blood flow, and should not be interpreted as increased cerebral tissue oxygenation.

In the December 2017 issue of the journal, Sørensen et al. [20] published a very elegant and complex interventional study in which they examined the effects of a vasoconstrictor (i.e. phenylephrine) on peripheral muscle oxygenation (SmO_2_) obtained with NIRS in 17 healthy volunteers. They also measured oxygenation of the skin and tibial bone as well as arm blood flow with Doppler ultrasonography and cardiac preload by transthoracic echocardiography and diameter changes of the inferior vena cava (IVC). The volunteers received phenylephrine to increase their MAP by about 30%, both during supine position and head-up tilt to simulate central hypovolemia. The macrohemodynamic effects of phenylephrine (increases in MAP, vascular resistance and stroke volume as well as decreases in heart rate, cardiac output and IVC collapsibility indicating improved cardiac filling and reduced fluid responsiveness) were in accordance with expectations and previous results [21, 22]. Regarding tissue oxygenation, phenylephrine significantly increased SmO_2_ while decreasing oxygenation at the skin and tibial level. While head-up tilting per se decreased SmO_2_, phenylephrine restored SmO_2_ completely to baseline level under this condition. The authors conclude that phenylephrine redistributed blood flow from the extremities towards the central compartment, and speculate that the paradoxical increase in SmO_2_ was caused by constriction of the venous part of the muscular vessels, changing the local ratio between oxygenated and deoxygenated blood within the muscle. This postulated mechanism was questioned in a letter by Grocott [23], who also added the possible explanation that phenylephrine may have augmented venous return (and thus cardiac output and muscle oxygen delivery). In their response, Sørensen et al. [24] quote data from a follow-up study where they could show that phenylephrine actually increased SmO_2_ by augmenting local oxy-hemoglobin concentration while decreasing that of de-oxyhemoglobin.

## Feasibility of NIRS monitoring in pediatric patients

While many studies focus on the association of NIRS-derived regional or cerebral oxygen saturation and perioperative outcome, or focus on other important issues such as the agreement of measurement methods, the actual time-related feasibility of obtaining regional oxygen saturation values is infrequently investigated. Yet, under some circumstances the speed with which measurements can be obtained is equally important as, for instance, its reliability. Recently, this issue was addressed by Ziehenberger et al. in the June 2018 issue of the journal [25] in term and preterm neonates directly after birth, and the time was counted how quickly cerebral oxygenation values could be obtained upon arrival at the resuscitation table. The authors demonstrated that generally it was possible to have cerebral oxygenation values within one minute, which potentially allows swift recognition and treatment of impaired cerebral perfusion in a critical time period of the newborn. Nevertheless, the clinical impact of such quick and early measurements has yet to be determined in future trials.

Additionally, bilateral placement of the NIRS sensors on the forehead of pediatric patients can be challenging, due to the minimal size required for a (pediatric) sensor, which must contain a light-emitting and usually two receiving optodes with a certain distance in between them, the anatomical restrictions (size) of the pediatric patient, and the need for an additional sensor for measuring the depth of anesthesia. Also, the frontal sinus behind the bow ridge in the skull might impair the accuracy of NIRS measurements on the forehead since it is filled with (non-conducting) air, limiting light propagation. In order to optimize sensor location (avoiding the frontal sinus), Kim and coworkers (October 2018) [26] performed a retrospective observational study in 203 pediatric patients aged 3–17 years who had undergone brain computed tomography, and measured the frontal sinus height, shape and pneumatization in relation to patient age. As expected, the frontal sinus height increased with age from around 6–21 mm, and it was diamond-shaped. Pneumatization of the frontal sinus was not visible in 78% of the younger patients (3–5 years old) and expands to almost 80% of patients at 6–8 years of age. Based on the correlation between age and sinus size the authors developed a simplified age-based method of sensor placement which allows to avoid the sinus in at least 90% of patients. They recommend placing the sensor 1, 2 or 3 cm above the superior orbital rim in children younger than 6 years, between 6 and 10 years, and above 10 years, respectively. This information is useful to increase the reliability, stability, accuracy, and feasibility of non-invasive NIRS monitoring in children.

Another feasibility study of pediatric NIRS monitoring was published by Hamrin et al. [27] in the same issue of the journal. The authors placed NIRS sensors both on the forehead and the abdomen in 38 children (mostly younger than 6 months) who had to undergo ground and air transport and prospectively examined the reliability of the measurements and data storing under these challenging circumstances. The collected data during transport were cleared for artefacts and smoothened by a noise-reduction algorithm. This way, reliable data were obtained in about 98% and 96% from the cerebral sensors and in 100% and 98% for the abdominal sensors during air and ground transport, respectively. Both cerebral and abdominal measurements decreased with increasing altitude during air transport. The authors concluded that NIRS measurements proved feasible both during ground and air transport for both cerebral and abdominal measurement sites in critically ill pediatric patients. Hence, NIRS measurements may give insight in physiological processes during transport and serve as a useful complement to existing monitoring during inter-hospital transports, not only in this particular group of fragile patients.

## Assessing oxygen delivery and maintenance at the (sub-)cellular level

In recent years, more attention has been paid to finding ways to obtain clinically useful information on tissue oxygen delivery and maintenance processes at the cellular and subcellular level. This has facilitated the development of non-invasive optical devices that are destined for the use to monitor tissue oxygenation at the bedside. At the microcirculatory level, this is commonly done by measurements of variables such as total vessel density (TVD), perfused vessel density (PVD) and proportion of perfused vessels (PPV). At the subcellular level, oxygen tension and consumption are being measured in mitochondria to assess cellular oxygen levels and utilised levels of oxygen in cells. As already addressed, local and systemic factors regulate blood flow by modulating precapillary sphincters, depending on metabolic needs of the tissue. The heterogeneity of perfusion is low in healthy individuals, however, the microcirculation has become a particular focus in pathophysiological conditions over the last decades as endothelial dysfunction at the organ level has been linked to sepsis and microcirculatory alterations have been observed to be more prominent in non-survivors than in survivors [28]. Therefore, hand-held microscopes (HVMs) have been developed and recently introduced into the clinic to assess the microcirculation and the second consensus paper on how to perform this was recently published [29]. The latest generation of HVM devices is based on incident dark-field (IDF) imaging and has improved optical lenses and high-resolution computer-controlled image sensors compared to the previous generation that is based on side-stream dark field (SDF) imaging. This improvement results in the identification of up to 20–30% more small vessel compared to SDF [30]. HVMs are most commonly applied sublingually due to the easy access to this site. Additionally, measurements in other places, i.e. the peritoneum after laparotomy, are emerging and compared to sublingual findings [31].

Important advances have been made in recent time in order to bring HVMs to the bedside of critical ill patients, however, issues relating to reliability and feasibility of their clinical application persist as demonstrated by Damiani et al. [32] and Carsetti et al. [33]. The study by Damiani et al. in the October 2017 issue of this journal shows how video quality influences the evaluation of sublingual microcirculation in critically ill patients. The importance of the video quality assessment (carried out according to Massey and Shapiro [30]) was highlighted by the observation that poor video quality can be linked to a lower degree of microvascular density and perfusion, suggesting that poor video quality may result in misleading conclusions about the state of the microcirculation. Microvascular perfusion paradoxically was reported to be lower in conscious patients (degree of consciousness assessed by means of Glasgow Coma Scale) than in unconscious patients (sedated and/or mechanically ventilated, degree of unconsciousness measured by dose of propofol administered), which the authors attribute to involuntary tongue or head movements by the fully-conscious patient that lack in unconscious patients and directly affect video quality of all assessed categories. Despite experience in use or sufficient training of all operators, video quality was acceptable in only 56% of all videos. Even the principle investigator, who has extensive experience, obtained only acceptable quality in 40% of all video triplets (three videos at the same time point in order to avoid bias). This study evaluated data obtained with the SDF technology and others have shown that IDF imaging is superior, however, the issues raised by Damiani et al. persist with the newest generation of HVMs and need to be considered by users.

The work by Carsetti et al. [33] in the August 2017 issue of this journal illustrates the second major issue that we are facing: the feasibility as well as the utility of the microcirculatory assessment is diminished by it being time-consuming and operator-dependent and thus, there is an urgent need for a reliable automated analysis software. Carsetti et al. compare the automatic analysis software CytoCamTools 1.7.12 (CC) to the most commonly used manual software Automated Vascular Analysis (AVA) 2.2. in terms of TVD of PVD and PPV of small vessels (< 20 µm), as well as a semi-quantitative (AVA) or quantitative (CC) assessment of the type of flow present. Results are shown as coefficients of correlation and mean bias and Carsetti et al. claim the correlation of the TVD results to be acceptable. However, the study has an intrinsic issue as it compares two software packages that rely on different methods to obtain results of flow-related variable, and thus, any comparison or correlation of results is disputable as pointed out by the accompanying editorial comment [34]. Therefore, even though an automated analysis software is desperately needed, it also needs to be sufficiently validated and interchangeable flow-variables are necessary to do so.

A different, potentially complementary approach for obtaining information about oxygen maintenance at the tissue level is presented by Ubbink et al. [35] and was published in the December 2017 in this journal. Their method allows a closer look at the actual site of oxygen consumption, namely the mitochondria, the so-called powerhouse of the cell. The COMET (Cellular Oxygen METabolism) device has been developed for the use in clinic and is based on the Protoporphyrin IX-triplet state lifetime technique (PpIX-TSLT), which provides an estimation of mitochondrial oxygen levels based on delayed fluorescence signals detected following stimulation of a component of the heme biosynthesis pathway. Measurements are carried out in the epidermis of the sternum as this is easily accessible and the heme synthesis pathway is not active in the mitochondria located there. The authors report an oxygen-consumption measurement made in a healthy volunteer, which shows an oxygen disappearance rate of 6.3 mmHg s^−1^ upon occlusion of the area of interest and a quick oxygen recovery. Further, in a patient undergoing neurosurgery, the immediate effect of the hypertension medication clonidine on the mitochondrial oxygen tension was observable with the COMET but not with the device O_2_C, which is used to assess the oxygen saturation of hemoglobin at the venous end of the capillaries as well as microcirculatory blood flow [36]. Further validation of the device regarding correlation of oxygen levels measured in the epidermis and in locations that are directly affected in the critical ill patient are required, however, the COMET appears to be a promising tool to obtain additional data on oxygen tension and consumption by evaluating subcellular oxygen levels.

## Conclusion

This end of year summary on monitoring—and provocation—of the microcirculation and tissue oxygenation shows that multiple study activities are going on in this evolving research field. It also shows that the methods involved (particularly NIRS and handheld videomicroscopy) have found their way from animal research to clinical applications. Nevertheless, more evidence has to be created showing that these new methods actually help improving patient`s outcome before they will find their place as routine clinical monitoring tools.
